# Regulatory mechanism and expression level of PRPS2 in lung cancer

**DOI:** 10.1111/1759-7714.15302

**Published:** 2024-05-12

**Authors:** Ying Meng, Hua Zhang, Mingling Xu, Zhenzhen Chen, Lei Wei

**Affiliations:** ^1^ Department of Oncology Zibo Central Hospital Zibo China; ^2^ Department of Pulmonary and Critical Care Medicine Zibo Central Hospital Zibo China

**Keywords:** apoptosis, lung cancer, proliferation, PRPS2

## Abstract

**Background:**

Lung cancer, with high morbidity and mortality, is the commonest respiratory system neoplasm, which seriously endangers the life safety of patients. In this study, the effect of PRPS2 on cell progression was preliminarily investigated.

**Methods:**

Immunohistochemical staining, western blot and reverse transcription‐quantitative polymerase chain reaction (RT‐qPCR) were performed to verify the expression level of PRPS2 in lung cancer. Lung cancer cell lines with stable downregulation of PRPS2 were constructed in A549 cells and NCIH460 cells. The function of PRPS2 silencing on the proliferation ability was verified by the EdU and cell colony formation experiment. Scratch and transwell tests were conducted to verify the role of PRPS2 silencing on the migratory and invasive ability of cells. The impact of PRPS2 silencing on cell apoptosis and cell cycle was verified by flow cytometry test. The effects of PRPS2 silencing on apoptosis‐associated proteins were assessed by western blot assay. The function of PRPS2 silencing on tumor growth in vivo was studied through xenograft tumor experiment.

**Results:**

In comparison with normal tissues, PRPS2 was upregulated in lung cancer tissues. PRPS2 knockdown notably hindered the migratory ability, invasive ability and proliferation, but accelerated cell apoptosis. In vivo experiments confirmed that PRPS2 silencing blocked the growth of transplanted tumors.

**Conclusion:**

In lung cancer, PRPS2 silencing suppressed the malignant progression, indicating that PRPS2 might be a novel biomarker for lung cancer treatment and diagnosis.

## Graphical abstract

PRPS2 knockdown significantly accelerated the apoptosis of lung cancer cells, but inhibited the proliferation, migration and invasion of lung cancer cells in vitro and the growth of transplanted tumors in vivo.

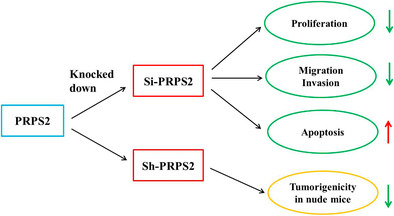

## INTRODUCTION

As the world's leading cancer killer, the incidence and mortality of lung cancer are increasing year by year.^1^ There are an estimated 800 000 deaths and 2 million new cases of lung cancer each year.^2^ Surgical resection is the preferred treatment for early stage lung cancer; however, about 20%–55% of lung cancer patients relapse after surgery.^3^ Unfortunately, most lung cancer patients are already at an advanced stage upon diagnosis, and the 5‐year overall survival rate is usually less than 20%.^4^ At present, it has been established that lung cancer is mainly caused by the interaction of environmental and genetic factors, but the specific molecular mechanism is still being studied. With the advent of the era of biological big data, especially the establishment of various malignant tumor network shared databases, more tumor treatment targets have been discovered, which undoubtedly promotes the rapid development of tumor targeted therapy.^5^, ^6^ Despite significant advances in targeted therapies for multiple genetic alterations, there is still a lack of effective therapeutic targets for lung cancer.

Phosphoribosyl pyrophosphate synthetase (PRPS) is a key rate‐limiting enzyme in nucleotide synthesis, containing three subtypes: PRPS1, PRPS2, and PRPS3.^7^ It has been found that PRPS1 and PRPS2 play important regulatory roles in different malignant tumors. PRPS1 is overexpressed in melanoma and accelerates tumor progression by promoting cell growth, metastasis, and suppressing cell apoptosis.^8^ PRPS2 is the target of myeloid proliferative oncogene (c‐Myc), which changes with c‐Myc expression.^9^ Previously, the expression levels of PRPS2 vary in different tumor tissues, and its related mechanism is preliminarily discussed. Song et al. reported that PRPS2 mutation is a biomarker for recurrence of childhood acute lymphoblastic leukemia.^10^ PRPS2 is markedly overexpressed in osteosarcoma and closely related to the recurrence rate and poor prognosis.^11^ Furthermore, the expression level of PRPS2 in prostate adenocarcinoma is sharply increased, and knocking down PRPS2 significantly promotes cell apoptosis and blocked cell multiplication.^12^ A previous study has confirmed that PRPS2 is enriched in the exosomes of lung cancer, and the M2 macrophage polarization mediated by exosomes PRPS2 enhances the cisplatin resistance.^13^ Nevertheless, the specific mechanism of PRPS2 on lung cancer cells needs to be further studied.

Herein, this study first examined the expression of PRPS2 in lung cancer tissues. Next, A549, and NCIH460 cells were selected to silence PRPS2 expression by RNA interference technology, aiming to explore the function of PRPS2 in the invasion, proliferation, migration, apoptosis and in vivo growth of lung cancer cells. Our study investigated the possible molecular mechanism of PRPS2 providing new evidence for the diagnosis and treatment of lung cancer.

## METHODS

### Cell lines

NCI‐H23, HCC827, A549, NCIH460 cells (human lung cancer cells) and BEAS‐2B cells (normal human lung epithelial cells) were obtained from Beyotime. RPMI 1640 medium containing 10% fetal bovine serum (FBS) and 1% penicillin/streptomycin was used to culture the cells. The incubator was set at 37°C, and 5% CO_2_.

### Cell transfection

PRPS2 siRNAs, obtained from Ribobio, were transfected into lung cancer cells with lipofectamine 3000 (Invitrogen). A lentiviral vector (Geneseed) was used to achieve stable knockout of PRPS2 in NCIH460 cells. NCIH460 cells were infected with PRPS2 shRNA lentivirus and empty viral vector. Subsequently, stable cell lines were screened with 5 μg/mL purinomycin. The knockdown effect of PRPS2 was detected by western blot.

#### Tissue specimens

With the approval of our hospital's ethics committee, 40 pairs of lung cancer tissues and adjacent tissues were collected after patients signed informed consent forms. All lung cancer patients were confirmed by tissue biopsy, and none received radiotherapy or chemotherapy or other antitumor therapy before surgery. None of the patients had serious medical diseases or other malignant tumors.

#### Immunohistochemical (IHC) staining

The paraffin sections were placed in boiling water bath of ethylenediaminetetraacetic acid (EDTA) solution for 15 min and then removed to cool naturally. At room temperature, goat serum was added to the tissues for 30 min. The first antibody was added to the tissues and incubated overnight at 4°C. At room temperature, the second antibody was added for 20 min. 3,3'‐diaminodbenzidine (DAB) developing solution was added for dyeing and hematoxylin was added for redyeing. After being sealed with neutral glue, the tissues were observed with a microscope.

#### Cell colony formation experiment

The cells were inoculated in a six‐well plate and incubated in a carbon dioxide incubator for 14 days. After adding formaldehyde for 20 min, staining cells were achieved by adding crystal violet for 30 s. Using a microscope (Olympus), the number of cell clusters greater than 50 cells was counted.

#### Western blot assay

The total protein was obtained by lysing the cells with radioimmunoprecipitation assay (RIPA) lysate. The gel film was then transferred to polyvinylidene fluoride (PVDF) membrane, after electrophoresis with SDS‐PAGE gel (12%). After sealing with 5% skim milk for 2 h, primary antibody was added for incubation (anti‐PRPS2,1:1000; anti‐GAPDH, 1:2000; anti‐cleaved caspase‐3, 1:500; anti‐Bax, 1:1000; anti‐Bcl‐2, 1:2000, all from Abcam) at 4°C. The next day, the second antibody was added to the PVDF membrane for 1 h. After adding ECL A solution and B solution, exposure development was performed using gel imager.

#### 5‐ethynyl‐2'‐deoxyuridine EdU assay

The cells (5 × 10^4^ cells/well) of logarithmic growth stage were inoculated into a 96‐well plate. The cells were cultured with 100 μL EdU medium for 2 h, and then 20 μL 4% paraformaldehyde was added. Subsequently, 50 μL glycine was added to each well for decolorization. The cells were permeated with 0.5% Triton X‐100 for 20 min and then stained with Hoechst 33342. EdU positive cells were observed under fluorescence microscopy (Olympus).

#### Flow cytometry (FCM)

The cells were inoculated into a 96‐well plate overnight at 37°C. After centrifugation at 4°C for 5 min, the cells were collected and resuspended according to the apoptosis kit instructions. After staining with annexin V‐FITC and PI reagents, the reaction was carried out at room temperature and away from light for 15 min. Flow cytometry was used to detect cell apoptosis in each group.

After digestion, frozen ethanol (75%) was added at 4°C overnight. Subsequently, after cell centrifugation, RNase A solution was added for 30 min. PI solution was then added to the cells and they were incubated at 4°C for 15 min away from light. Flow cytometry was then used for cell cycle analysis.

#### Reverse transcription‐quantitative polymerase chain reaction (RT‐qPCR)

Total RNA was extracted from cells using TRIzol/chloroform, and reverse‐transcribed into cDNA by using a Prime‐Script kit. Using cDNA as template, the PCR reaction system was prepared with STBR Premix ExTaq reagent. 2^−△△Ct^ was used to display the mRNA relative expression. GAPDH F: 5′‐TGCACCACCAACTGCTTAGC‐3′, R: 5′‐GAGGGGCCATCCACAGTCTTC‐3′; PRPS2 F: 5′‐AGCTCGCATCAGGACCTGT‐3′, R: 5′‐ACGCTTTCACCAATCTCCACG‐3′.

#### Transwell assay

For cell invasion, the transwell chamber was first placed in a 24‐well plate, and 50 μL Matrigel was spread in the center of the Transwell chamber. Then, 100 μL cell suspension was added to the upper chamber, and 600 μL Dulbecco's modified Eagle medium (DMEM) was added to the lower chamber. The 24‐well plates were placed in a 5% CO_2_ incubator at 37°C for 48 h. After removing the matrix glue and residual cells, the chamber was stained in 0.1% crystal violet staining solution. Using an inverted microscope (Olympus), cell counts and photographs were taken for each sample. For cell migration, the experimental steps were the same as above, except for not embedding Matrigel.

### Scratch test

The cells were digested with trypsin, and 500 μL cell suspension (5 × 10^5^ cells/well) was added to a 24 well plate. The cells were cultured in a 5% CO_2_ incubator at 37°C for 16–24 h. When the cells grew to confluent, a uniform scratch was made with the tip of a 10 μL pipette. After 24 h, photographs were taken under the microscope and the healing rate of scratches was calculated.

#### Xenograft tumor experiment in nude mice

Ten BALB/C nude mice (4‐week‐old) were divided randomly into sh‐NC and sh‐PRPS2 groups. Then, 200 μL cell suspension (5 × 10^6^ cells/mL) was inoculated into the armpits of nude mice. Tumor formation in nude mice was observed after inoculation. After tumor appeared, tumor volume was measured in vitro with vernier calipers once a week. The mice were killed by CO_2_ inhalation after 4 weeks. The tumors were removed to measure volume, weighed and photographed. After photographing, the tumors were fixed with 10% paraformaldehyde overnight for immunohistochemical test. All animal experiments were approved by our hospital's Animal Research Ethics Committee and conducted in accordance with relevant guidelines and regulations.

#### Statistical analysis

The data are expressed as mean ± SD and were analyzed using SPSS22.0 software. A *t* test was carried out to compare measurement data among groups. Graph Pad Prism 8.0 software was used for statistical mapping. *p*‐values < 0.05 were considered statistically significant.

## RESULTS

### Expression of PRPS2 in lung cancer

**FIGURE 1:**
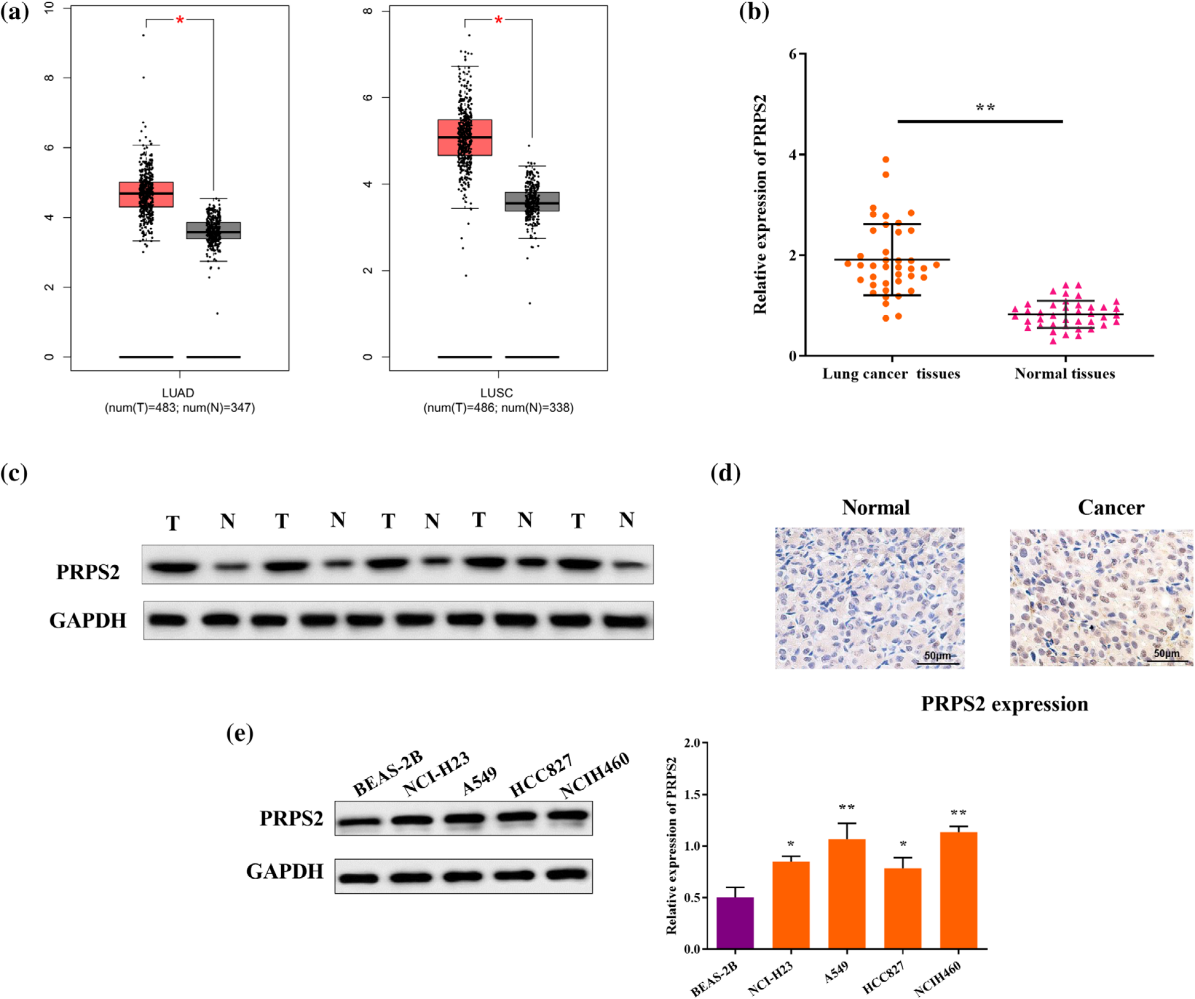
Expression of PRPS2 in lung cancer. (a) The expression levels of PRPS2 in lung adenocarcinoma (LUAD) and lung squamous cell carcinoma (LUSC) from the TCGA dataset. (b) PRPS2 mRNA expression in lung cancer tissues and normal tissues. (c) PRPS2 protein expression in lung cancer tissues and normal tissues. (d) Representative images of PRPS2 IHC stain of lung cancer and normal tissues. (e) PRPS2 protein expression in lung cancer tissues and normal tissues. *p < 0.05; **p < 0.01.

According to TCGA, mRNA levels of PRPS2 in lung cancer were clearly higher than in normal tissues (Figure 1a). According to RT‐qPCR and western blot, we also found the increased expression of PRPS2 was in lung cancer tissues (Figure 1b,c). Next, compared with normal tissues, immunohistochemical (IHC) analysis confirmed that PRPS2 expression in lung cancer tissues was higher (Figure 1d). Furthermore, compared with BEAS‐2B cells, PRPS2 was higher expressed in NCI‐H23, A549, HCC827, SKMES1, NCIH460 cells (Figure 1e). Here, the results suggested that PRPS2 may be involved in the malignant progression of lung cancer.

### PRPS2 silencing inhibited cell growth

**FIGURE 2:**
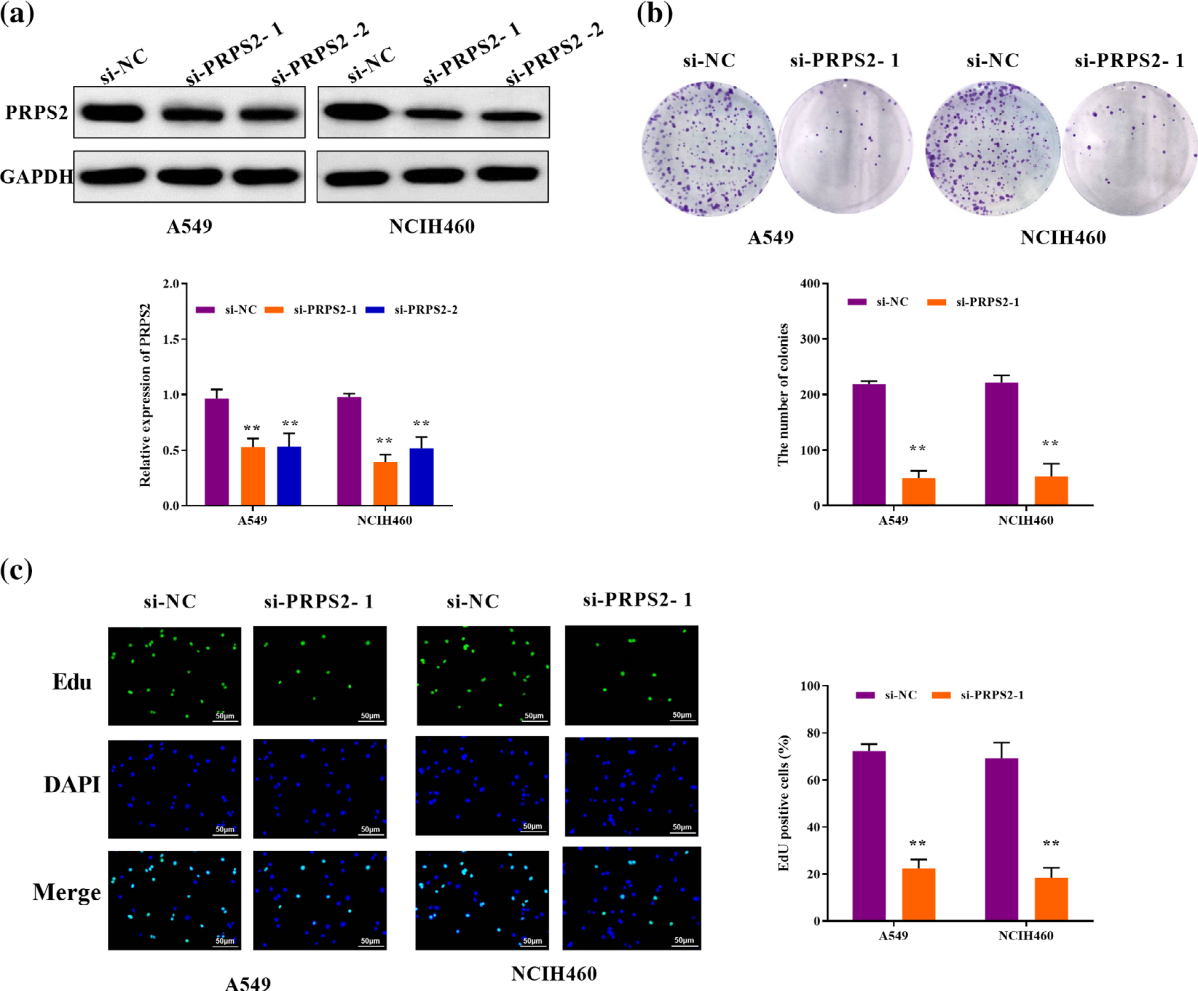
PRPS2 silencing inhibited cell growth. (a) The protein expression of PRPS2 was detected by western blot after transfection. (b) The relative efficiency of clonal formation in cells transfected with PRPS2 silencing. (c) The proliferation capacity of cells transfected with PRPS2 silencing was evaluated by EdU assay. **p < 0.01.

As described in the western blot assay, both si‐PRPS2‐1 and si‐PRPS2‐2 knocked down PRPS2 expression (Figure 2a). We selected A549 and NCIH460 cells transfected with si‐PRPS2‐1 for follow‐up experiments. The function of PRPS2 on cell growth was verified by EdU and cell colony formation assay. As described in Figure 2b, compared to the control group, the number of cell colonies in the PRPS2 silencing group was lower. Similarly, PRPS2 silencing reduced the EdU positive cells (Figure 2c). Therefore, our experiments confirmed that PRPS2 silencing repressed the cell growth.

### PRPS2 silencing inhibited cell migration and invasion

**FIGURE 3:**
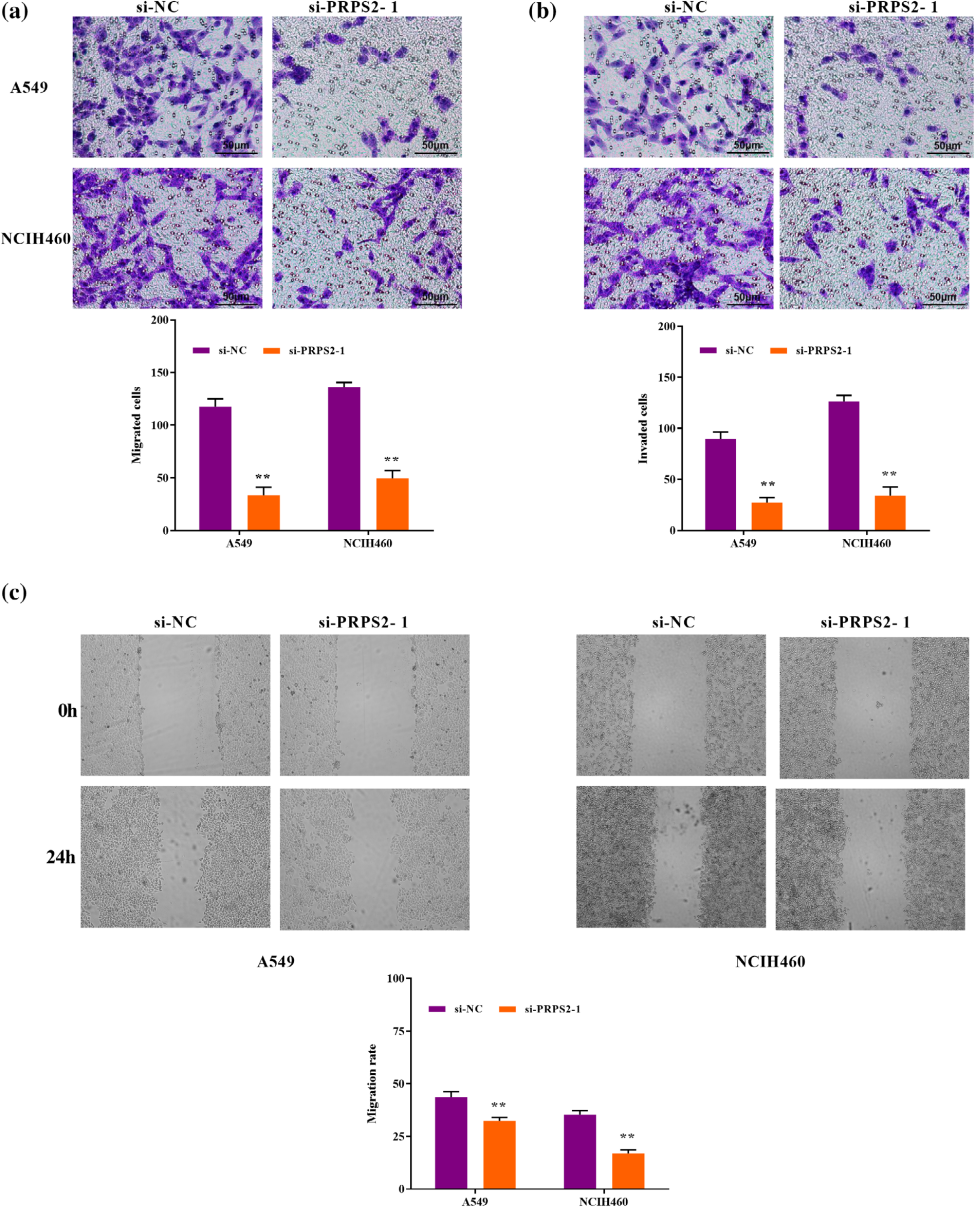
PRPS2 silencing inhibited cell migration and invasion. Transwell assay was performed to determine the migration (a) and invasion (b) abilities of A549 and NCI‐H446 cells with PRPS2 silencing. (c) Scratch test was performed to measure the migration ability of A549 and NCI‐H446 cells with PRPS2 silencing. Magnification: ×200. Scale bars = 100 μm. **p < 0.01.

Next, in the group transfected with PRPS2 silencing, the number of migrated cells was sharply decreased (Figure 3a). Furthermore, in comparison with the control group, PRPS2 silencing reduced cell invasion in A549 and NCIH460 cells (Figure 3b). A scratch test showed that the migration rate in the PRPS2 silencing group was dramatically reduced compared with the control group (Figure 3c). Together, the data indicated that PRPS2 silencing blocked cell migratory and invasive ability.

### PRPS2 silencing promoted cell apoptosis

**FIGURE 4:**
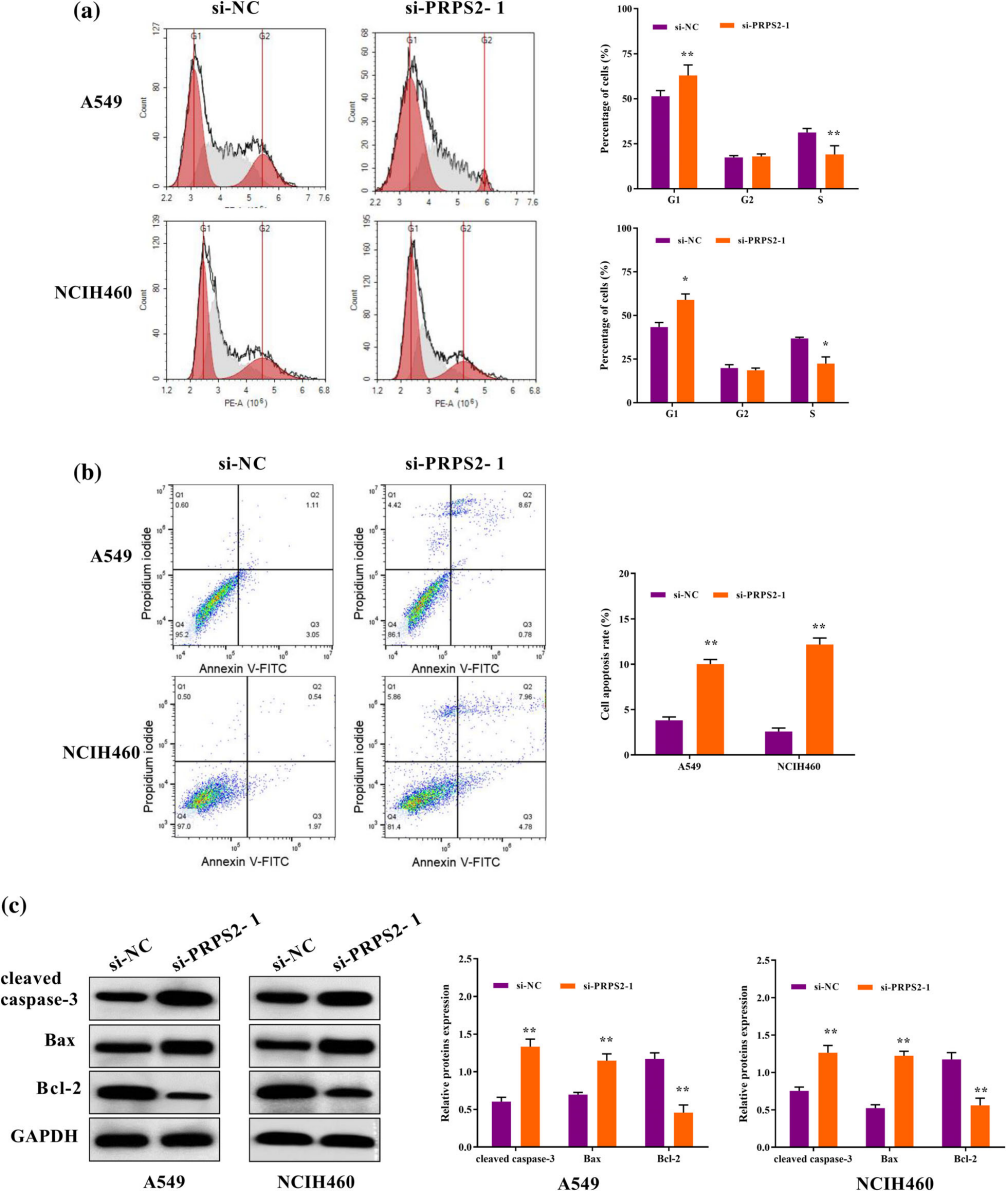
PRPS2 silencing promoted cell apoptosis. The cell cycle (a) and cell apoptosis (b) of cells transfected with PRPS2 silencing was evaluated by flow cytometry (FCM). (c) The expression of apoptosis‐related proteins was detected by western blot assay. *p < 0.05; **p < 0.01.

Inhibition of PRPS2 increased the DNA content of G1 phase cells and decreased the S phase cells (Figure 4a). The apoptosis rate of cells transfected with PRPS2 silencing was clearly increased (Figure 4b). In the PRPS2 silencing group, Bax and cleaved caspase‐3 were notably elevated, and Bcl‐2 level was decreased (Figure 4c). The data indicated that low expression of PRPS2 accelerated apoptosis of lung cancer cells.

### PRPS2 silencing suppresses tumor growth in vivo

**FIGURE 5:**
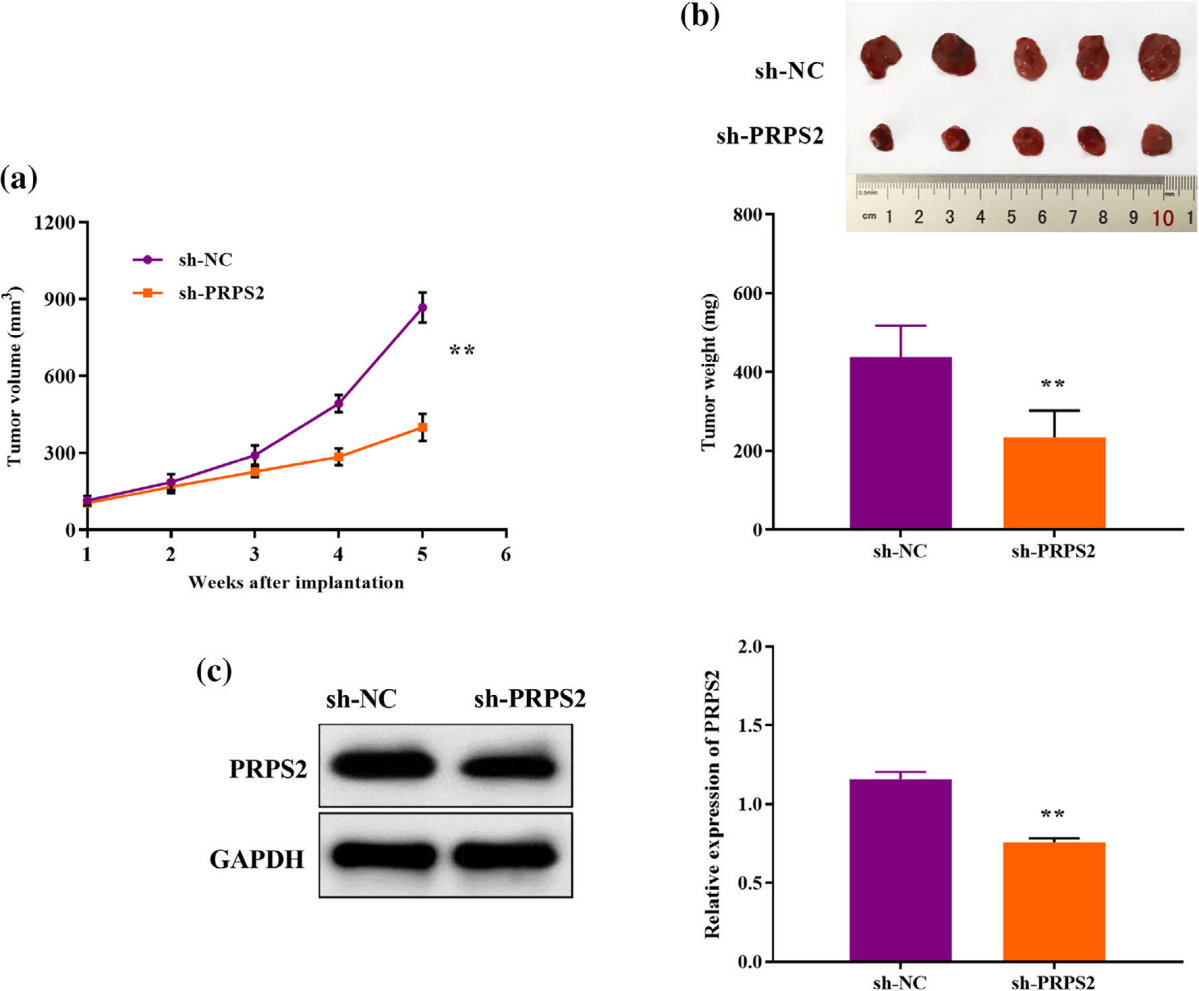
PRPS2 silencing suppresses tumor growth in vivo. (a) Tumor volume was measured every 7 days after injection. (b) At 35 days after injection, xenograft tumors were removed and weighed. (c) PRPS2 protein levels in xenograft tumors were detected by western blot. **p < 0.01.

Compared with the control group, the tumor volume in sh‐PRPS2 group decreased over time (Figure 5a). On day 35, the tumor growth index of the control group was higher than that of the PRPS2 knockout group (Figure 5b). Moreover, the expression of PRPS2 in sh‐PRPS2 group was lower than that in control group. (Figure 5c). The results proved that knocking down PRPS2 restrained the growth of lung cancer tumors in vivo.

## DISCUSSION

Lung cancer has become the most dangerous cancer for human health due to its high morbidity and mortality.^14^ Most patients die from recurrence or metastasis, and there is no effective treatment.^15^ Herein, the pathogenesis of lung cancer is still unclear, and molecular studies are needed to find effective therapeutic targets.

PRPS2 is a key coupling protein and rate‐limiting enzyme in nucleic acid biosynthesis, mainly involved in the synthesis of purine nucleotides and pyrimidine nucleotides and the synthesis of some nuclear coenzymes, histidine and tryptophan.^9^, ^16^ In melanoma, PRPS2 has been shown to be a direct target of c‐Myc, and knocking down PRPS2 leads to a reduction of dNTP and delays cell cycle progression.^17^ PRPS2 has been confirmed to be notably overexpressed in prostate cancer^12^ and cervical cancer,^18^ suggesting that PRPS2 contributes to cancer progression. PRPS2 is reported to enhance the resistance of lung cancer to DDP,^13^ but there are few reports on the lung cancer cell progression. In the present article, it was found that the expression of PRPS2 was higher than that of normal tissues, so we speculated that PRPS2 might participate in lung cancer progression. We interfered with PRPS2 expression in A549 and NCIH460 cells and found that the proliferation activity of silencing PRPS2 cells was obviously reduced. Furthermore, after knocking down PRPS2, the apoptosis rate was accelerated. We subsequently discovered that cells transfected with PRPS2 silencing could not transition from the G1 phase to S phase, resulting in G1/S arrest. This is due to the lack of synthesis of purine nucleotides, which is difficult to meet the rapid growth of tumor cells. Our study confirms that knocking down PRPS2 suppresses the malignancy of lung cancer by blocking cell proliferation and promoting cell apoptosis.

Metastasis is the biological characteristic of malignant tumors, and aggressive malignant tumors can not only grow at the primary site, but also spread to surrounding tissues.^19^, ^20^ The primary cause of death in cancer patients is tumor cell metastasis, which is the most important biological feature of malignant tumors.^21^ Most patients with lung cancer will develop metastases, which seriously reduce the survival rate of patients.^22^ In this study, PRPS2 silencing was found to notably reduce cell invasive ability and migratory ability. Likewise, in cervical cancer, PRPS2 silencing repressed the invasion and migration.^23^ Similar findings showed that the metastasis ability of colorectal cancer cells was enhanced after PRPS2 overexpression.^24^ However, it is noteworthy that knockdown of PRPS2 suppresses cell proliferation but does not affect cell migration in neuroblastoma.^25^ Therefore, PRPS2 knockdown retards lung cancer progression by inhibiting cell migration and invasion.

Nevertheless, there were still some shortcomings in the study of the role of PRPS2 in lung cancer progression. For example, there was a lack of other functional validation experiments, and immunohistochemical detection of PRPS2 in mouse tissue sections. In addition, the molecular signaling pathways through which PRPS2 is involved in the occurrence and development of lung cancer are still unclear, and further studies are needed.

In conclusion, this study found that PRPS2 silencing suppressed the malignant behavior of lung cancer cells by restraining cell invasion, migration, proliferation, and promoting cell apoptosis, which has relevant clinical significance and provides new ideas for the study of molecular mechanism in lung cancer.

## AUTHOR CONTRIBUTIONS

Ying Meng and Hua Zhang contributed to the conception of the study. Mingling Xu and Zhenzhen Chen contributed significantly to the data analysis and study preparation. Ying Meng performed the data analyses and wrote the study. Zhenzhen Chen and Lei Wei helped perform the analysis with constructive discussions. All authors have read and approved the final study.

## FUNDING INFORMATION

This research received no external funding.

## CONFLICT OF INTEREST STATEMENT

The authors declare no conflict of interest.
